# A novel CAV derived cell-penetrating peptide efficiently delivers exogenous molecules through caveolae-mediated endocytosis

**DOI:** 10.1186/s13567-018-0513-2

**Published:** 2018-02-13

**Authors:** Gaowei Hu, Wenlv Zheng, Ao Li, Yaru Mu, Mingyu Shi, Tuofan Li, Haitao Zou, Hongxia Shao, Aijian Qin, Jianqiang Ye

**Affiliations:** 1 Key Laboratory of Jiangsu Preventive Veterinary Medicine, Key Laboratory for Avian Preventive Medicine, Ministry of Education, College of Veterinary Medicine, Yangzhou University, Yangzhou, 225009 Jiangsu China; 2 Jiangsu Co-innovation Center for Prevention and Control of Important Animal Infectious Diseases and Zoonoses, Yangzhou, 225009 Jiangsu China; 3 Joint International Research Laboratory of Agriculture and Agri-Product Safety, The Ministry of Education of China, Yangzhou University, Yangzhou, 225009 Jiangsu China; 4 Institutes of Agricultural Science and Technology Development, Yangzhou University, Yangzhou, 225009 Jiangsu China

## Abstract

Cell-penetrating peptide (CPP) is a promising cargo for delivering bioactive molecules. In this study, the N terminus of VP1 from chicken anemia virus, designated as CVP1, was found to carry enriched arginine residues with α-helix. By confocal imaging, flow cytometry and MTT assay, we identified CVP1 as a novel, safe and efficient CPP. CVP1-FITC peptide could entry different types of cells tested with dose dependence, but without cytotoxic effects. Compared with TAT-FITC peptide, the CVP1-FITC peptide showed much higher cell-penetrating activity. Moreover, CVP1 could successfully deliver β-glycosidase, poly (I:C) and plasmid into HCT116 cells. Inhibitors and temperature sensitivity analysis further indicated that the cell-penetrating activity of CVP1 was based on ATP-dependent and caveolae-mediated endocytosis. All these data demonstrate that CVP1 has efficient cell-penetrating activity and great potential for developing a novel delivery vector.

## Introduction

Cell-penetrating peptide (CPP) is an active cargo for delivering bioactive molecules into cells [1]. Generally, CPPs are composed of 5–30 amino acids (aa) with enriched lysine or arginine residues, and divided into three classes including amphipathic CPP, cationic CPP and hydrophobic CPP [2, 3]. Among the three classes, HIV (Human Immunodeficiency Virus)-TAT peptide (aa 47–57 of the trans-activator protein TAT from HIV), as classic cationic CPP, has been extensively studied and applied. As the pioneer of CPPs, HIV-TAT with 86 amino acids plays vital roles in the replication of HIV. It was reported that HIV-TAT contains enriched arginine and lysine residues with α-helical structure which play a key role in cell penetrating capacity [4, 5]. At present, TAT is used as an ideal tool to deliver the apoptin for cancer therapy [6, 7]. However, TAT peptide sequence contains a motif that is recognized and cleaved by furin so that its stability and cell penetrating capacity would be removed during the process to deliver exogenous cargoes [8]. Thus, a more efficient novel cell penetrating peptide needs to be developed.

In this study, the sequence and structure analysis first revealed that the N-terminus of VP1 protein (designated as CVP1) from chicken anemia virus (CAV) carried a α-helical structure consisting of enriched arginine residues similar with HIV-TAT. The efficient cell-penetrating activity of CVP1 was identified through confocal imaging, flow cytometry and MTT assay. Moreover, the peptide CVP1 could efficiently deliver both protein and nucleic acids into cells.

## Materials and methods

### Reagents

Hoechst 33342 purchased from Beyotime (Shanghai, China). β-Galactosidase, potassium ferricyanide, potassium ferrocyanide, magnesium chloride and X-gal were obtained from Sangon Biotech (Shanghai, China). Chlorpromazine (CPZ, a clathrin-mediated endocytosis inhibitor [9], methylated-β-cyclodextrin (MβCD, a caveolae-mediated endocytosis inhibitor, a cholesterol depletion agent [10]), 5-(*N*-ethyl-*N*-isopropyl) amiloride (EIPA, a macropinocytosis inhibitor, selectively blocks the Na^+^/H^+^ antiporter [11]) and LY294002 (a macropinocytosis inhibitor, a strong phosphatidylinositol-3 kinase inhibitor [12]) were obtained from Aladdin Industrial (Shanghai, China).

### Design and synthesis of peptides

Amino acid sequences of the VP1 from different isolates of CAV were collected from GenBank. VP1 sequences from 13 CAV isolates (GenBank Accessions No. AFC89546.1, AJR29322.1, ACR19999.1, ACR20005.1, AAC55985.1, AJR29324.1, ACT31535.1, ABR10397.1, AAU87871.1, AAT47125.1, AHA41686.1, CCF79482.1, AIL25648.1) were aligned using MultAlin server [13]. Prediction and evaluation of potential CPPs from VP1 protein N-terminus (CVP1) was carried out using CellPPD server [14]. Structural predictions of the CVP1 and TAT (aa 47–57) were made using the PEP-FOLD3 server [15]. The predicted structure was displayed using the PyMoL software. The mutant of CVP1 (designated as Mut-CVP1) served as the negative control, and all arginine residues in CVP1 were substituted with alanine (A) or glycine (G) in Mut-CVP1 (Table 1). Meanwhile, the HIV-TAT peptide was used as a positive control [4, 5]. All synthetic peptides were labeled with FITC at the N-terminus. Peptides were synthesized by F-moc solid-phase peptide synthesis from GenScript Biotech (Nanjing, China) with ≥ 95% purity. The sequences of the synthesized peptides were shown in Table 1.

**Table 1:** **Sequences of CVP1, TAT peptide and analogs used in the study**

No.	Peptide name	Sequence	Molecular weight^a^
1	CVP1	FITC-MARRARRPRGRFYAFRRGR	3110.56
2	Mut-CVP1	FITC-MAAGAGAPAGAFYAFGAGA	2131.36
3	HIV-TAT	FITC-YGRKKRRQRRR	2062.38

^a^Molecular weight of FITC-labeled peptides.

### Cell culture

293T cells, HCT-116 cells, 3T3 cells, MDCK cells and MSB1 cells were maintained in our laboratory and were grown in Dulbecco’s modified Eagle’s medium supplemented with 10% heated-inactivated fetal bovine serum (FBS) and 1% penicillin–streptomycin solution at 37 °C and 5% CO_2_.

### Confocal microscopy

Firstly, 2.0 × 10^5^ of 293T cells, HCT-116 cells, 3T3 cells or MDCK cells were seeded onto glass cover slides in 24-well plates and cultured for 12 h. The cells were then washed with phosphate buffered saline (PBS) and incubated with 5 µM of FITC-labeled CVP1, TAT and Mut-CVP1 peptides in 500 µL serum-free medium for 30 min. The concentration-dependent intake of CVP1 peptide in HCT-116 cells was then evaluated. In brief, after incubation of cells with FITC-CVP1 peptide for 30 min, cells were washed for three times and stained with Hoechst 33342 (5 µg/mL) for 10 min. Finally, cells were washed three times with PBS and imaged using confocal laser scanning microscope (Leica, TCS SP8 STED and Germany) at 60× magnification.

### Flow cytometry

The comparison of cell-penetrating efficiency of peptides in HCT116 cells was tested by incubating the cells with CVP1 or TAT peptides. After incubation, the cells were then washed three times with PBS and were further incubated with trypsin for 5 min to remove peptides that did not penetrate the cell membrane [16]. The cells were washed twice by centrifugation and finally resuspended in 400 µL PBS for assaying of cellular fluorescent intensity by flow cytometry (BD FACSAria SORP, Becton–Dickinson, USA). A total of 10 000 events were recorded. Data was obtained and analyzed using Flowjo software.

### MTT assay

Effect of CVP1 on the viability of cells was evaluated by MTT assay as described previously [17].

### Cell internalization pathway

The mechanism of uptake of CVP1 in HCT116 cells was investigated by means of physical and chemical inhibition of endocytosis. Incubation of peptide and cells at 37 or 4 °C were treated to measure the influence of temperature on peptide internalization (used as a physical means of inhibition of endocytosis). HCT116 cells cultured in 12-well plates were pretreated for 30 min with inhibitors: 30 µM chlorpromazine (a clathrin-mediated endocytosis inhibitor, which prevents the assembly of clathrin-coated pits at the inner surface of the cell membrane), 5 mM methylated-β-cyclodextrin (a caveolae-mediated endocytosis inhibitor, a cholesterol depletion agent), 10 µM 5-(*N*-ethyl-*N*-isopropyl) amilorid (a macropinocytosis inhibitor, which selectively blocks the Na^+^/H^+^ antiporter) and 50 µM LY294002 (a macropinocytosis inhibitor, a strong phosphatidylinositol-3 kinase inhibitor) as previously described [18, 19]. The cells were further incubated with the peptide for 50 min and then analyzed by flow cytometry. To investigate the effect of incubation time, the HCT116 cells were incubated with CVP1 at 37 °C for different time periods.

### Analysis of the ability of CVP1 to deliver the β-galactosidase

Protein delivery ability of CVP1 was assessed using β-galactosidase enzyme as previously described [20, 21]. In brief, CVP1 and Mut-CVP1 at a concentration of 20 µM were non-covalently complexed with β-galactosidase (1 µL, 1 µg/µL) enzyme respectively. The mixtures were incubated for 30 min at room temperature (RT) for complex formation. The peptide-enzyme complex was added to the HCT116 cells with Opti-MEM media (100 µL) and the cells were incubated for 1 h at 37 °C. After incubation, complete media (150 µL) was added to the cells, and further incubated for 90 min at 37 °C. The cells were washed thoroughly with PBS to remove extracellular peptide-enzyme complex and fixed in PBS containing 1% formaldehyde and 0.2% glutaraldehyde for 15 min at RT. The cells were washed subsequently three times with PBS and stained with X-gal staining buffer (40 mM potassium ferricyanide, 40 mM potassium ferrocyanide, 20 mM magnesium chloride, 2 mg/mL of X-gal in PBS) for 90 min at 37 °C. Finally, the cells were analyzed under a phase-contrast microscope (Olympus) at 200× magnification and imaged.

### Evaluation of the ability of CVP1 to deliver plasmid

The linking of DNA with CVP1 was made by the charge interaction. To confirm the formation of the peptide and plasmid complexes, the CVP1 peptide (5 μM) and plasmid DNA (200 ng) were diluted in 10 µL Opti-MEM and co-incubated at 37 °C for 45 min, and then agarose gel retardation assay was performed as described previously [22, 23]. The transfection experiment was conducted to test the transfection efficiency of CVP1-DNA on HCT116 cells as described previously [24]. Briefly, 1 μg of plasmid DNA and 40 µM of CVP1 peptide or TAT peptide were diluted in 50 µL Opti-MEM, respectively and incubated at RT for 30 min. The lipofectamine 2000 (Invitrogen, USA) was used as a positive control, following the manual provided by the manufacture. Finally, the cells were observed at 48 h post-transfection by fluorescence microscope at 40× magnification. Meanwhile, the fluorescence intensity was measured by Flow cytometry as described previously.

### Analysis of the ability of CVP1 to deliver poly (I:C)

To analyze the ability of CVP1 to delivery poly(I;C), the CVP1 (10 µM) was first mixed with the poly (I:C) (5 µg) for 45 min at 37 °C for generating the complex. And then, the HD11 cells were incubated with peptide-poly (I:C) complexes, peptide (10 µM) only or poly (I:C) (5 µg) with transfecting reagent. After 6 h of incubation, the cells were collected and total RNA extracted using total RNA purification kit (Axygen, USA). 1 µg of RNA was reverse transcribed into cDNA with a reverse transcription reagent kit (Takara, Japan). The relative mRNA level of IFN-β was detected by Real-time PCR.

### Statistical analysis

All experiments were conducted three times in triplicate. Data is presented as mean ± S.D. Statistical analyses were performed using SPSS software (Version 16; SPSS, Inc, Chicago, IL, USA). The statistical differences in efficiency of internalization between the CVP1 and TAT, capacity of delivering the plasmid DNA and RNA and CVP1 internalization at different temperature were calculated by independent-samples T test. Differences were considered significance for *P* < 0.05.

## Results

### CVP1 carried enriched argnine residues with α-helix

**Figure 1:**
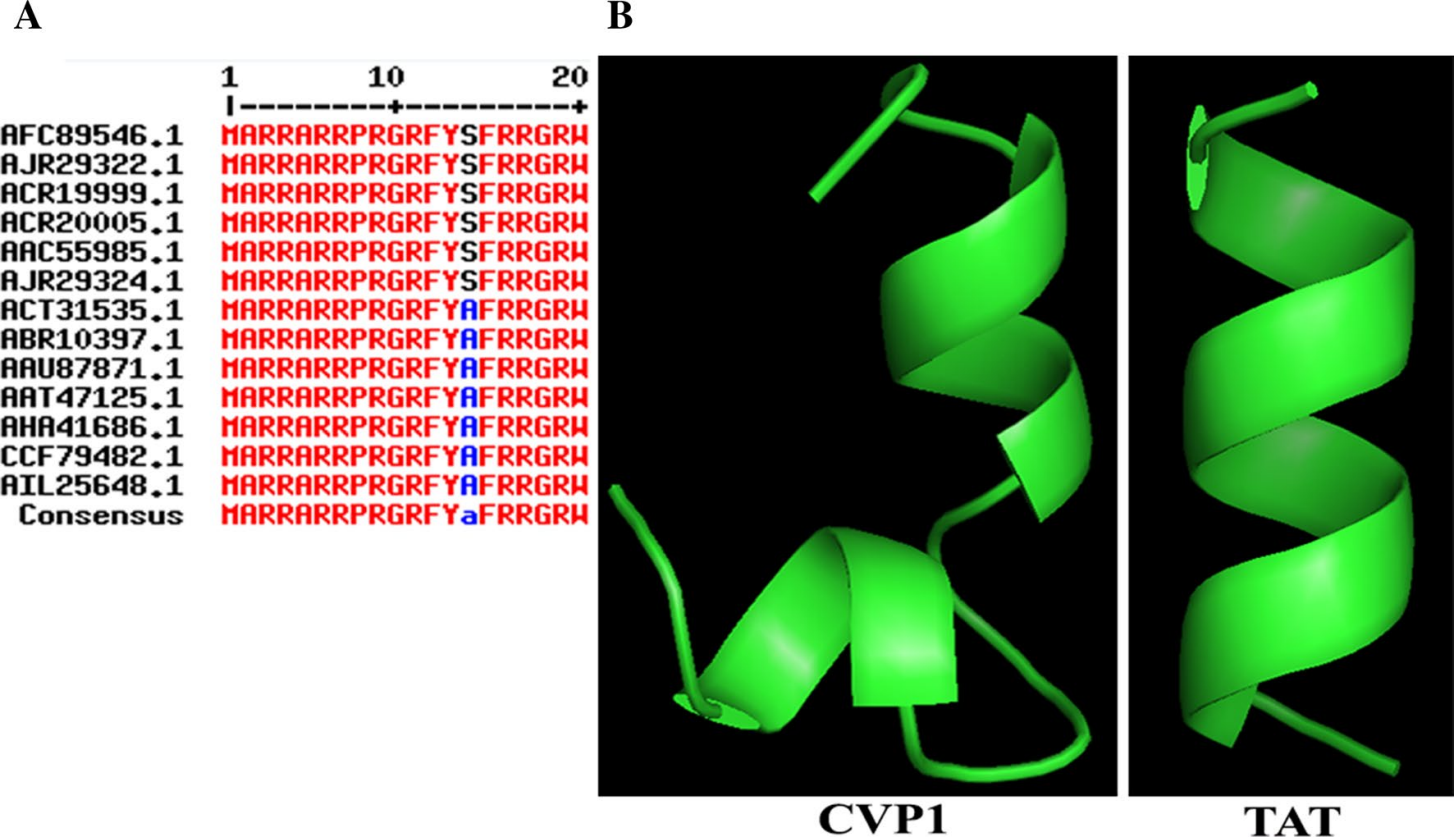
**Multi alignment of reference VP1 partial sequences and displaying the predicted structure of CVP1 and TAT by PyMOL™. A** The reference strains of CAV VP1 were aligned using MultAlin server. The red letters represent the identical amino acid residues among the different reference VP1 sequences. The blue or black letters represent the different amino acid residues. **B** Comparison of structure between CVP and TAT.

By the sequence alignment, we found that the N-terminus (aa 1–19) of the VP1 of CAV, designated as CVP1, was not only highly conserved, but also rich in arginine residues (Figure 1A). Since the cationic CPPs generally carry multiple basic amino acids, the CVP1 sequence was submitted to the CellPPD server to evaluate its potential cell-penetrating activity. In addition, the predicted space structure of CVP1 contains α-helix similar to TAT by using by PyMOL™ (Figures 1B and C). These data suggested that CVP1 had the potential to function as a CPP. To evaluate the cell-penetrating activity of the CVP1, three peptides listed in Table 1 were synthesized.

### CVP1 peptide had efficient cell-penetrating activity

**Figure 2:**
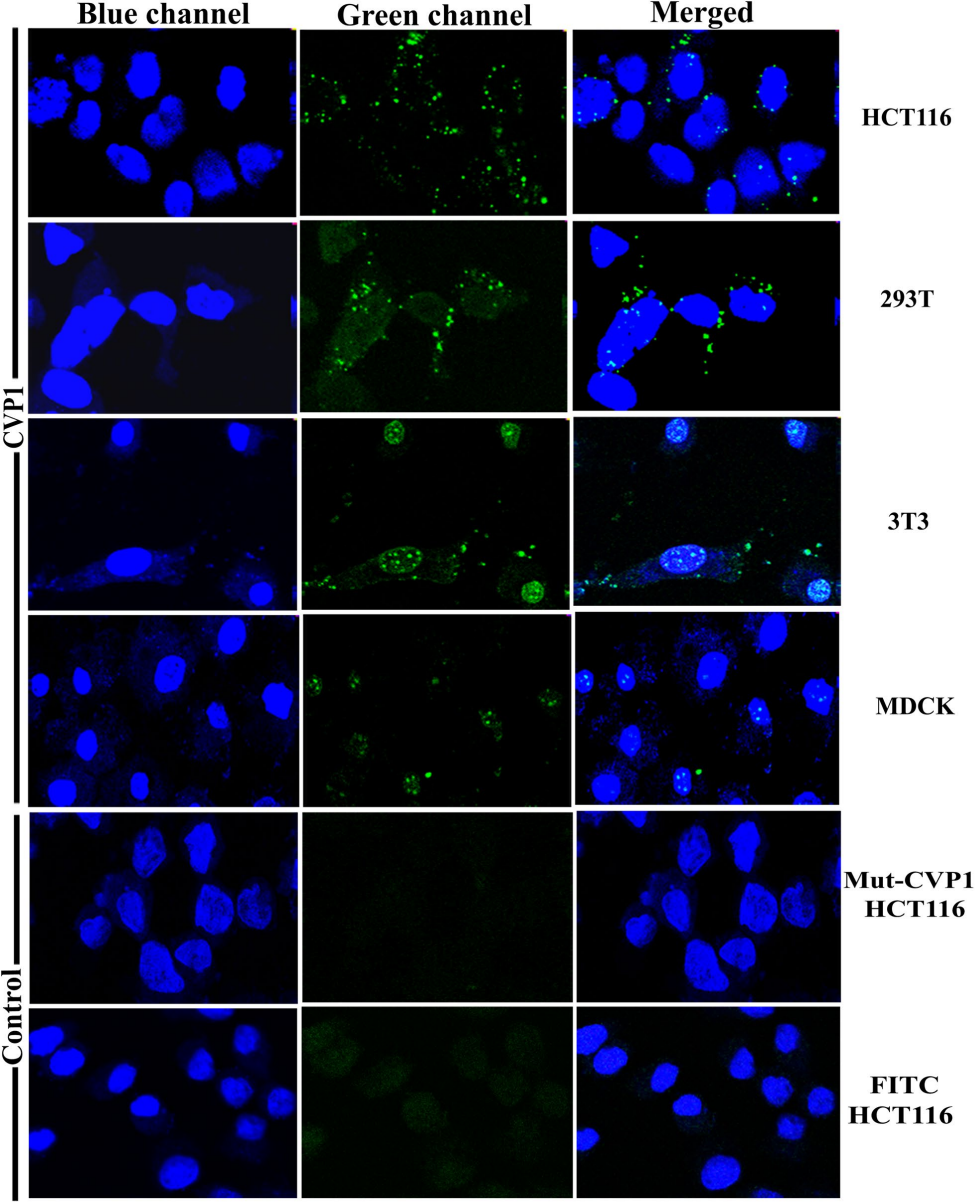
**The intracellular location of CVP1 peptide in different cell types and imaging by confocal microscopy.** HCT116, 293T, 3T3, MDCK cells were treated with 5 µM of CVP1 peptide for 30 min at 37 °C. The co-incubation of Mut-CVP1 and FITC dye with HCT116 cells served as the negative control. The nuclei was stained with Hoechst 33342 and presented the blue signal. The intracellular location of FITC-labeled CVP1 was indicated by the green signal. The pictures from left to right were blue channel, green channel and overlapped images, respectively.

**Figure 3:**
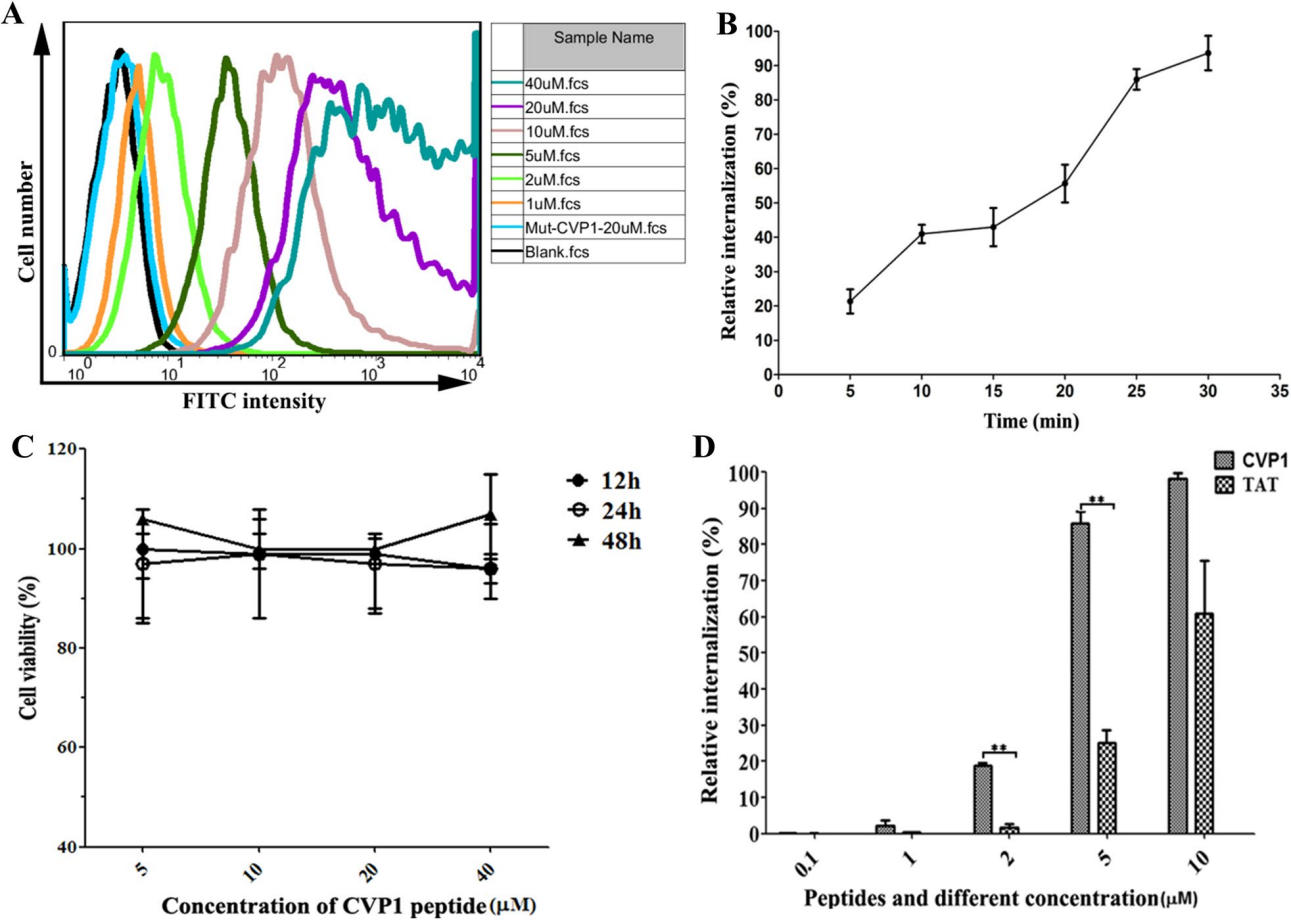
**The character of CVP1 peptide cell-penetrating activity. A** Analysis of the intake of CVP1 peptide in HCT116 cells at increasing concentration by flow cytometry. HCT116 cells were treated with different concentrations (1, 2, 5, 10, 20, 40 µM) of CVP1 or Mut-CVP1 peptide for 30 min at 37 °C. The data was shown using the FlowJo software. **B** HCT116 cells treated with the CVP1 peptide for the different incubation time points (5, 10, 15, 20, 25, 30 min) at a peptide concentration of 5 µM. **C** Percentage of viable HCT116 cells after treatment with CVP1 peptide at various concentrations (5, 10, 20, 40 µM) for 12, 24, and 48 h. **D** The cellular uptake efficiency of CVP1 and TAT peptide were evaluated by flow cytometry at 0.1, 1, 2, 5 and 10 µM. Single asterisk (*) and double asterisks (**) indicated *P* < 0.05 and *P* < 0.01, respectively.

To investigate the cell-penetrating activity and its pattern, the FITC-labeled CVP1 and Mut-CVP1 were incubated, respectively with different cells including HCT116, 293T, 3T3, MDCK at a final concentration of 5 µM for 30 min. As shown in Figure 2, the specific green fluorescence dots could be clearly observed in HCT116, 293T, 3T3 and MDCK cells treated with CVP1, but not with Mut-CVP1 and FITC dye only. And results of flow cytometry indicated that the cell-penetrating efficiency of CVP1 was dose-dependent (Figure 3A). The intensity of fluorescence in HCT116 cells was also increased as the incubation time was increased from 5 to 30 min (Figure 3B). The MTT assay showed that CVP1 was non-toxic to the cells even at a high concentration (40 µM) (Figure 3C). Notably, CVP1 showed more efficient cell-penetrating efficacy than TAT. The mean percent of relative internalization of CVP1 was 0.1, 2.1, 18.7, 86, 98% at 0.1, 1, 2, 5 and 10 µM, respectively, whereas that of TAT was 0.1, 0.4, 1.7, 25, 61, % respectively. All these data demonstrated that CVP1 peptide has efficient cell-penetrating activity with broad cell types.

### CVP1 efficiently delivered biomolecules into cells

**Figure 4:**
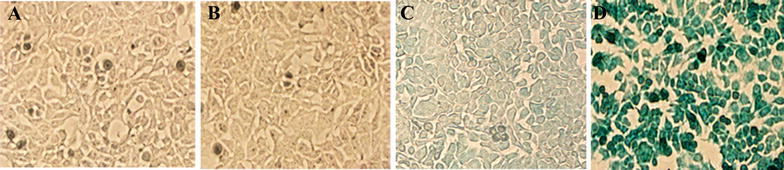
**The efficiency of CVP1 peptide delivering protein into HCT116 cells. A** HCT116 cells treated with β-galactosidase enzyme alone. **B** Cells treated with X-gal staining buffer alone. **C** Cells treated with non-covalent complex of Mut-CVP1 peptide and β-galactosidase enzyme. **D** Cells treated with non-covalent complex of CVP1 peptide and β-galactosidase enzyme.

**Figure 5:**
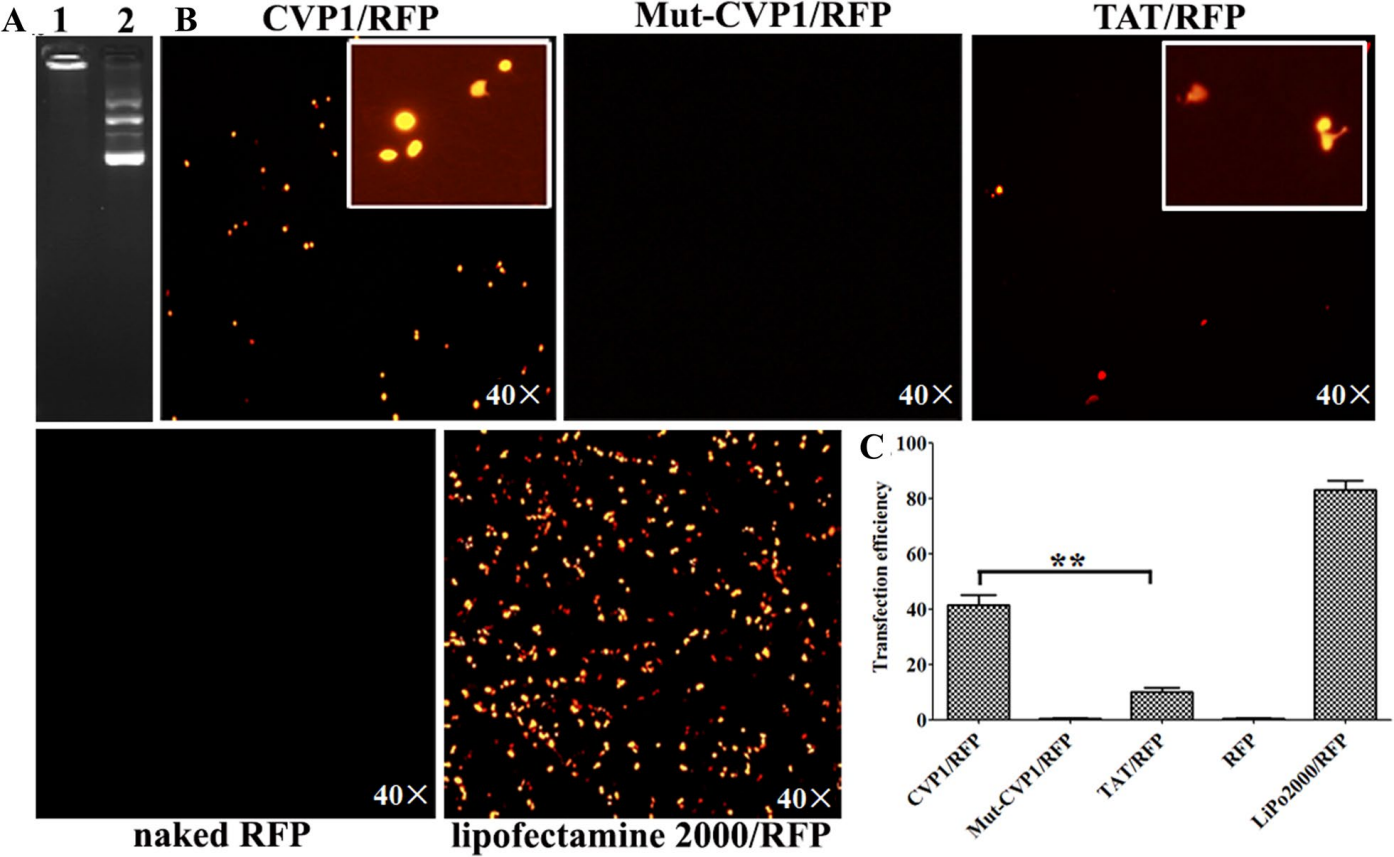
**The efficiency of CVP1 peptide delivering DNA into HCT116 cells. A** Detection of DNA and peptide complexes was conducted by electrophoresis and ethidium bromide staining. Lane 1: pCDNA3.1-RFP plasmid conjugated with CVP1 peptide; Lane 2: pCDNA3.1-RFP plasmid only; Fluorescence microscope images of the expression of the red fluorescence protein. The images obtained at a ×40 magnification. **B** Images of the CVP1/RFP complex, Mut-CVP1/RFP complex, TAT/RFP complex, pCDNA3.1-RFP only and transfection reagent lipofectamine 2000/RFP complex. **C** Quantification of the transfection efficiency using flow cytometry. Single asterisk (*) and double asterisks (**) indicated *P* < 0.05 and *P* < 0.01, respectively.

**Figure 6:**
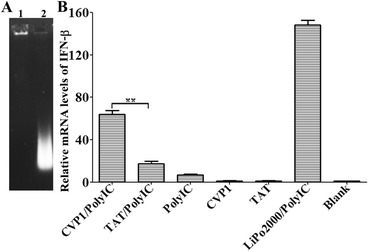
**Assaying the poly (I:C) and CVP1 complexes by gel retardation electrophoresis and detection the relative mRNA levels of IFN-β by real-time PCR. A** Testing the complex of poly (I:C) and CVP1 was conducted by electrophoresis and ethidium bromide staining. Lane 1: production of CVP1 peptide co-incubated with poly (I:C); Lane 2: poly (I:C) only; **B** The mRNA level of IFN-β obtained from the complexes of CVP1 peptide and TAT conjugated with poly (I:C), respectively, TAT and CVP1 peptide only, transfection reagent with poly (I:C) and blank cells. Single asterisk (*) and double asterisks (**) indicated *P* < 0.05 and *P* < 0.01, respectively.

To evaluate the capacity of CVP1 as a vector to deliver exogenous molecules into cells, β-galactosidase enzyme, plasmid and poly (I:C) linked with CVP1 was delivered into HCT116 cells, respectively. As shown in Figure 4, the cells treated with CVP1-enzyme showed strong blue color whereas cells treated with enzyme or X-gal substrate only did not show any blue color through X-gal staining (Figure 4). And the cells treated with Mut-CVP1-enzyme complex only showed very weak blue background. The DNA binding capacity of the CVP1 was confirmed by agarose gel-shift assay, in which pCDNA3.1-RFP plasmid conjugated with CVP1 showed much lower migration speed than naked plasmid (Figure 5A). The cells treated with CVP1-pCDNA3.1-RFP showed strong fluorescence whereas no fluorescence was observed in cells treated either with the naked DNA or with the Mut-CVP1-pCDNA3.1-RFP complexes. Furthermore, the CVP1/RFP showed significantly higher signal of fluorescence than the TAT/RFP sample. The results suggest that the CVP1 has superior transfection efficacy compared with TAT peptide (Figures 5B and C). For RNA delivery, the CVP1-poly (I:C) complex was incubated with HD11 cells and the efficacy of the delivery was measured by detection of IFN-β mRNA level. As shown in Figure 6, the CVP1 could conjugate with the poly (I:C) to form a complex and cells treated with the CVP1/poly (I:C) complexes significantly increased IFN-β mRNA expression when compared with the cells treated with the naked CVP1 or the poly (I:C) only. In addition, the capacity of delivering the poly (I:C) was higher than the TAT. All these data demonstrated that CVP1 was able to be used as carrier with high efficiency to deliver exogenous protein, DNA and RNA into cells.

### Cell-penetrating activity of CVP1 mediated by caveolae endocytosis pathway

**Figure 7:**
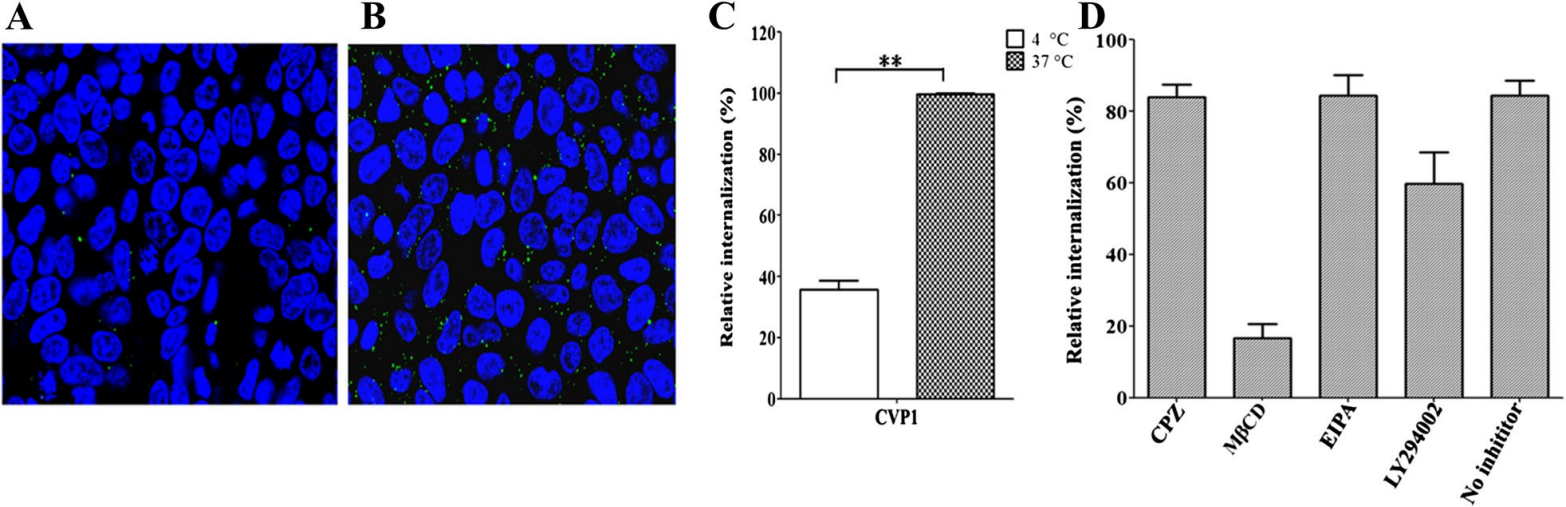
**Effect of temperature and endocytosis inhibitor on CVP1 internalization.** Confocal images of HCT116 cells incubated with CVP1 (10 µM) at 4 °C (**A**) and 37 °C (**B**); **C** the flow cytometry data for the effect of temperature were indicated as mean ± SD. Single asterisk (*) and double asterisks (**) indicated *P* < 0.05 and *P* < 0.01, respectively. **D** The effect of inhibitors on endocytosis is shown as mean ± SD.

To investigate the cell-penetrating mechanism of CVP1 peptide, four kinds of endocytosis inhibitors chlorpromazine (CPZ), methylated-β-cyclodextrin (MβCD), 5-(*N*-ethyl-*N*-isopropyl) amiloride (EIPA) and LY294002 were used. Flow cytometry data showed that MβCD significantly reduced the cellular intake of CVP1, whereas CPZ and EIPA were unable to inhibit its cellular intake (Figure 7). However, LY294002 only slightly blocked the cellular intake of CVP1. In addition, the flow cytometry analysis showed that cell-penetrating activity of CVP1 peptide was efficiently inhibited at 4 °C, but not at 37 °C. All these data indicated that the cell-penetrating activity of CVP1 was mainly mediated by the caveolae-mediated and ATP-dependent endocytosis pathway.

## Discussion

As classic cationic CPP, the HIV-TAT peptide has been applied as a promising tool to deliver biologically active cargoes into cells [25]. However, the TAT peptide sequence contains the motif (RKKR and RQRR) which could be digested by Furin. Therefore, TAT cell penetrating capacity and stability was affected by Furin [8]. In this study, a novel cell-penetrating peptide CVP1 based on the N terminus of CAV VP1 protein was identified. Our data showed that CVP1 not only had efficient cell-penetrating activity with different cell types, but also could be used as an efficient vehicle to deliver exogenous molecules into cells.

According to our data, the cell-penetrating activity of the CVP1 was much higher than that of TAT. The sequence with different amino acids and structure between CVP1 and TAT may contribute to the difference of the cell-penetrating activity [26, 27]. The TAT peptide has only one α-helix, while the CVP1 has two α-helices and additional arginine residues (Figure 1). The mutant of CVP1, Mut-CVP1, without arginine residues did not show any cell-penetrating activity, confirming the importance of these arginine residues for efficient cell-penetrating activity of CVP1 in agreement with another study published previously [28]. Moreover, compared with TAT, CVP1 displayed a larger hydrophilic and smaller hydrophobic face which could enhance its binding to the plasma membrane [29]. Taken together, these findings suggest that the CVP1 identified in this study could be as an alternative CPP for TAT in the future.

The efficacy of CVP1 peptide as a carrier for macromolecules was confirmed by efficient delivery of β-galactosidase and nucleotides (Plasmid and poly (I:C)) into cells. But the efficacy of delivering plasmid and poly (I:C) by CVP1 was not as equal as commercial transfection reagents. However, it should be noted that the CVP1 was conjugated with protein or nucleic acid by non-covalent interaction in current study. It is hypothesized that the fusion expression of CVP1 with foreign protein or the modified CVP1 could significantly increase the efficacy for delivering foreign molecules into cells. Based on the previous studies from TAT, the CCP with polymers or oligomers or branches could increase the delivering efficacy [9, 22, 24]. Similar studies with CVP1 are on-going. Currently, the exact uptake mechanism of CPPs is not clearly understood due to the component of CPP, cell types and cargoes [25]. Endocytosis plays an important role in the internalization of exogenous molecules. The endocytotic process includes caveolae-mediated endocytosis, clathrin-mediated endocytosis and macropinocytosis [30]. In order to determine which distinct endocytosis pathway was involved with CVP1, various endocytic inhibitors CPZ,MβCD,EIPA and LY294002 were employed. Through using the low temperature condition and endocytic inhibitors, we found that the CVP1 intake mainly depended on the caveolae-mediated endocytosis.

In summary, we identified a novel and safe cell-penetrating peptide derived from CAV VP1 protein, designated CVP1. The CVP1 peptide identified here not only could efficiently deliver exogenous molecules (including protein, DNA and RNA) into cells, but also showed higher cell-penetrating activity than TAT. Thus, our data provided a “proof of concept” that the CVP1 could be developed as an alternative CPP for TAT for more efficient delivery of drug or active molecules (such as apoptin and functional siRNA) into cells for therapeutic application in vivo.

## Abbreviations

CAV: chicken anemia virus
CPPs: cell-penetrating peptides
FITC: fluorescein isothiocyanate
RT: room temperature
FBS: fetal bovine serum
MDCK cells: madin-darby canine kidney cells
IFN-β: interferon β
